# The principle of maximum entropy and the probability-weighted moments for estimating the parameters of the Kumaraswamy distribution

**DOI:** 10.1371/journal.pone.0268602

**Published:** 2022-05-31

**Authors:** Amal Helu

**Affiliations:** Department of Mathematics, The University of Jordan, Amman, Jordan; The Research Institute at the Nationwide Children’s Hospital and the Ohio State University, UNITED STATES

## Abstract

Since Shannon’s formulation of the entropy theory in 1940 and Jaynes’ discovery of the principle of maximum entropy (*POME*) in 1950, entropy applications have proliferated across a wide range of different research areas including hydrological and environmental sciences. In addition to *POME*, the method of probability-weighted moments (*PWM*), was introduced and recommended as an alternative to classical moments. The *PWM* is thought to be less impacted by sampling variability and be more efficient at obtaining robust parameter estimates. To enhance the *PWM*, self-determined probability-weighted moments was introduced by (Haktanir 1997). In this article, we estimate the parameters of Kumaraswamy distribution using the previously mentioned methods. These methods are compared to two older methods, the maximum likelihood and the conventional method of moments techniques using Monte Carlo simulations. A numerical example based on real data is presented to illustrate the implementation of the proposed procedures.

## Introduction

A wide variety of methods have been developed to perform parameter estimation; see e.g. [1] for a discussion of some (frequently limited) sets of data. Common methods include, but are not limited to, the maximum likelihood method and the method of moments. The former is the most important method since it leads to efficient parameter estimators that are asymptotically normal. However, the maximum likelihood (*MLE*) method is not easy to obtain, highly computational and too sensitive to extreme values especially for small samples. Although *MLE* method is satisfactory for large samples, however, the final estimate is not always a global maximum because it can depend on the starting value. Moreover, the *MLE* method does not frequently lend itself to close form or easily manipulated algebraic expressions, even though it is known to provide asymptotically minimum variance, but not necessarily unbiased estimators.

The method of moment (*MOM*) is widely applied due to its relative ease of application. Moreover, the *MOM* can help to obtain a starting value for the numerical procedures involved in *MLE* estimation.

Other estimating methods have been developed as alternatives. Among these, the probability-weighted moments (*PWM*) which is a generalization of the classical moments of probability distribution. [2, 3] showed that the *PWM* can remove the ambiguity from the *MLE*. This method which was initiated by [4] constituted a leading alternative to the moment and *MLE* methods for fitting statistical distributions for which the inverse *F*(*x*) is analytically possible in a closed form; that is, if *X* is a random variable and *F* is the value of the cumulative distribution function of *X*, the value of *X* may be written as a function of *F*: *X* = *X*(*F*), which can be explicitly defined. [4, 5] showed that the *PWM* can derive simple expressions for the parameters for most of the distributions, including several for which parameter estimates are not readily obtained by using the method of *MLE* or conventional moments. In addition, the *PWM* method is thought to be less affected by sampling variability and is more efficient at producing robust parameter estimates from small samples [6].

In order to enhance the accuracy of the *PWM* [7] introduced the self-determined probability-weighted moment (*SD*-*PWM*) method. The *SD*-*PWM* method has the capability to more accurately account for the variation in the data sample, as well as the ability to consider any outliers that may be presented their. Moreover, *SD*-*PWM* method can reveal if a particular distribution is suitable for portraying the behavior of the sample data [7–9].

In case of scarce data, the principle of maximum entropy (*POME*) is used to generate least biased probability distribution that is appropriate for a wide range of applications such as hydrology frequency analysis, where a large amount of data is not available. [10] used entropy to quantitatively describe the uncertainty or information content of a random event *X*.

[11] developed the principle of maximum entropy (*POME*) as a tool for choosing some specific probability distribution from the set of feasible solutions. The chosen distribution maximizes the entropy function subjected to satisfying information constrains via the method of Lagrange multipliers. Hence, this distribution is consistent with the given information, but retains maximum uncertainty within the feasible domain and thus ensures the least biased [12]. Therefore, the parameters of the distribution can be obtained by achieving the maximum of the entropy function. [13] showed that for a given information such as mean, variance, skewness, etc., the distribution derived by *POME* would best represent *X*; implicitly, this distribution would best represent the sample from which the information was derived. Inversely, if it is desired to fit a particular probability distribution to a sample data, then *POME* can uniquely specify the constraints (or the information) needed to derive that distribution. The distribution parameters are then related to these constraints. An excellent discussion of the underlying mathematical rationale is given in [14, 15].

The Kumaraswamy distribution which is also known as a generalized beta distribution of the first kind is an extremely important and complex distribution used for risk analysis and reliability mechanisms with great success in biomedical and epidemiological research. The Kumaraswamy distribution is one of these distributions that is particularly beneficial for many natural phenomena whose outcomes have lower and upper bounds or bounded outcomes, such as individuals’ heights, test scores, atmospheric temperatures, economic and hydrological data.

**Fig 1:**
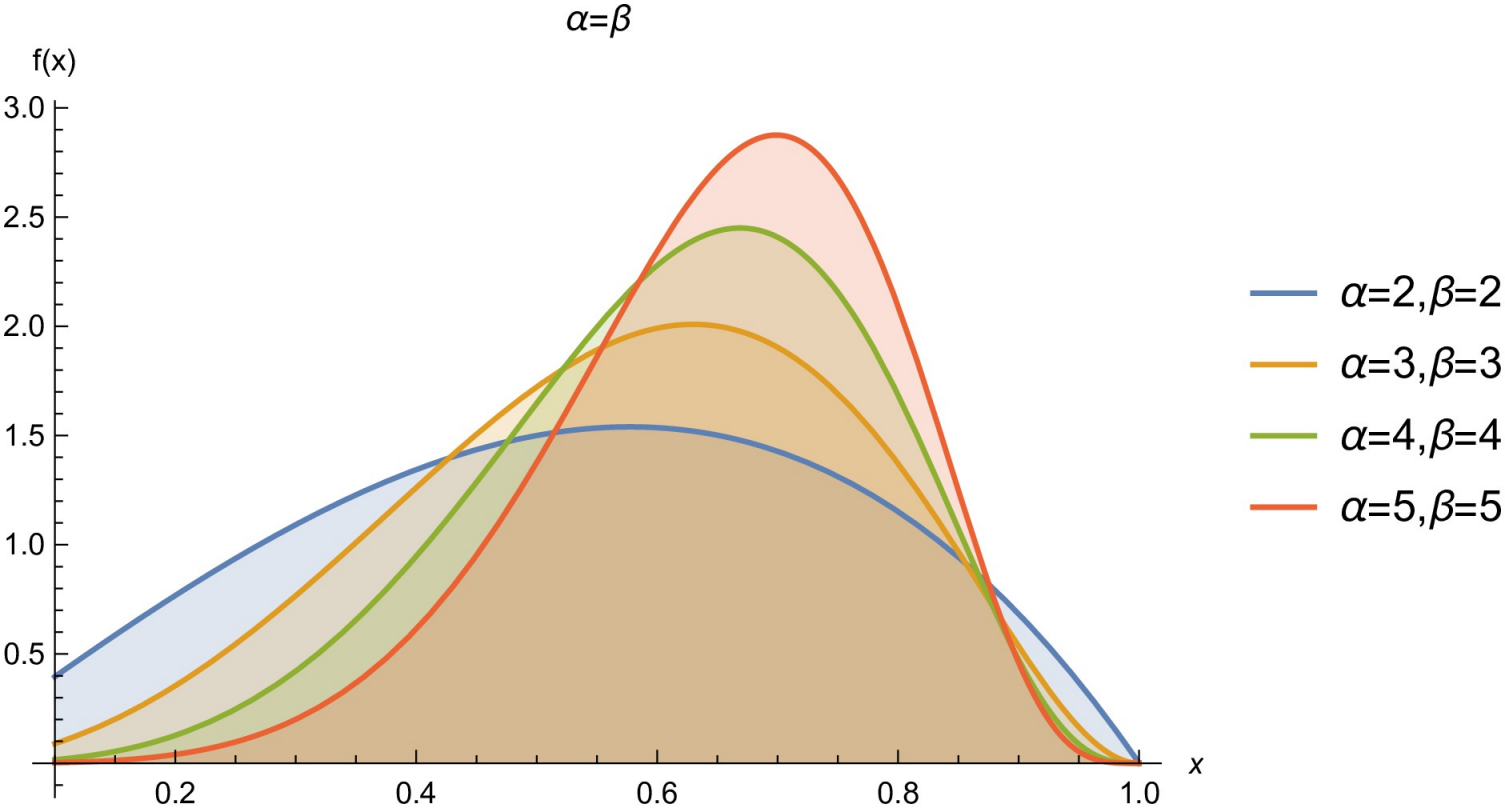
pdf of *Kum*(*α*, *β*), when *α* = *β*.

**Fig 2:**
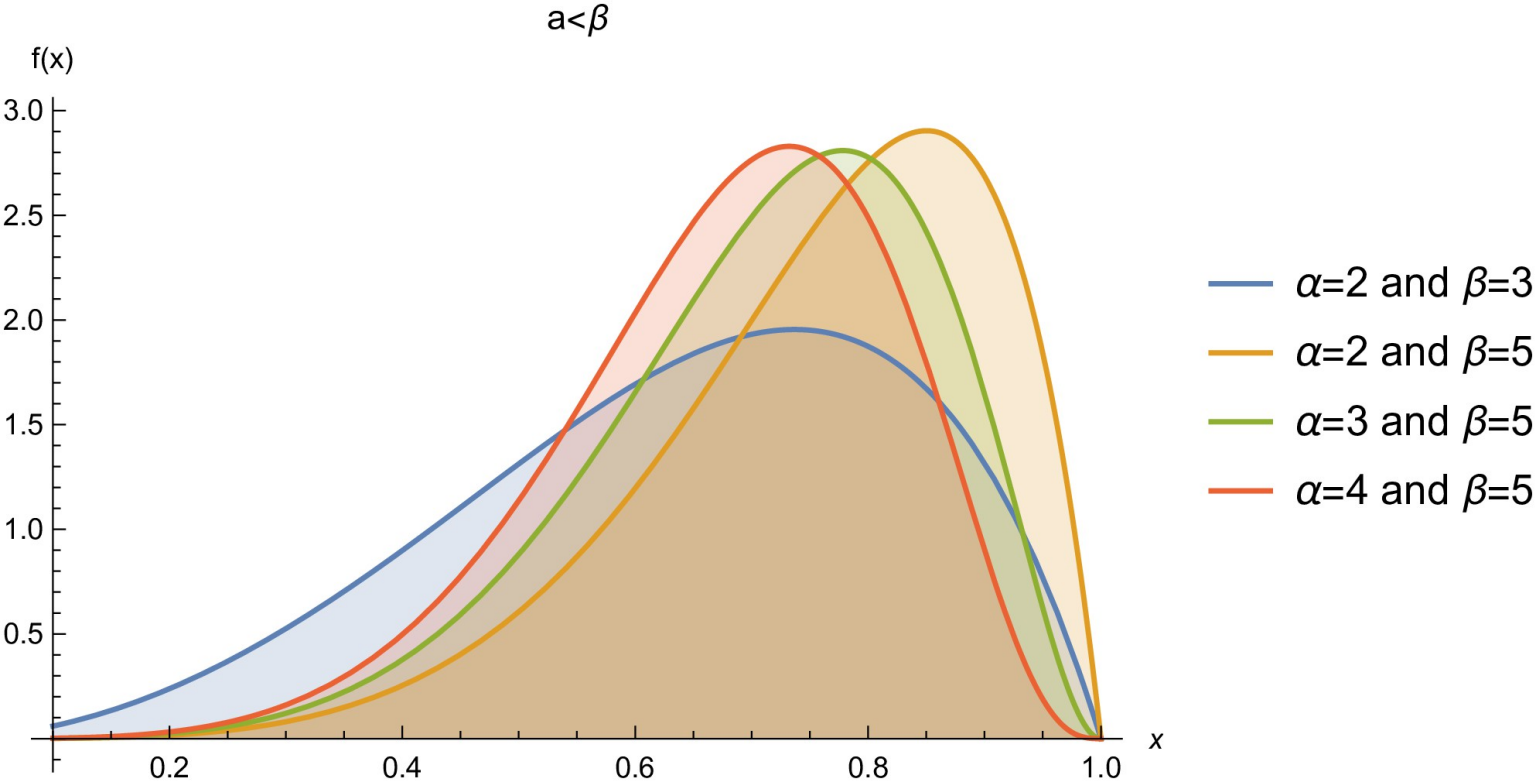
pdf of *Kum*(*α*, *β*), when *α* < *β*.

**Fig 3:**
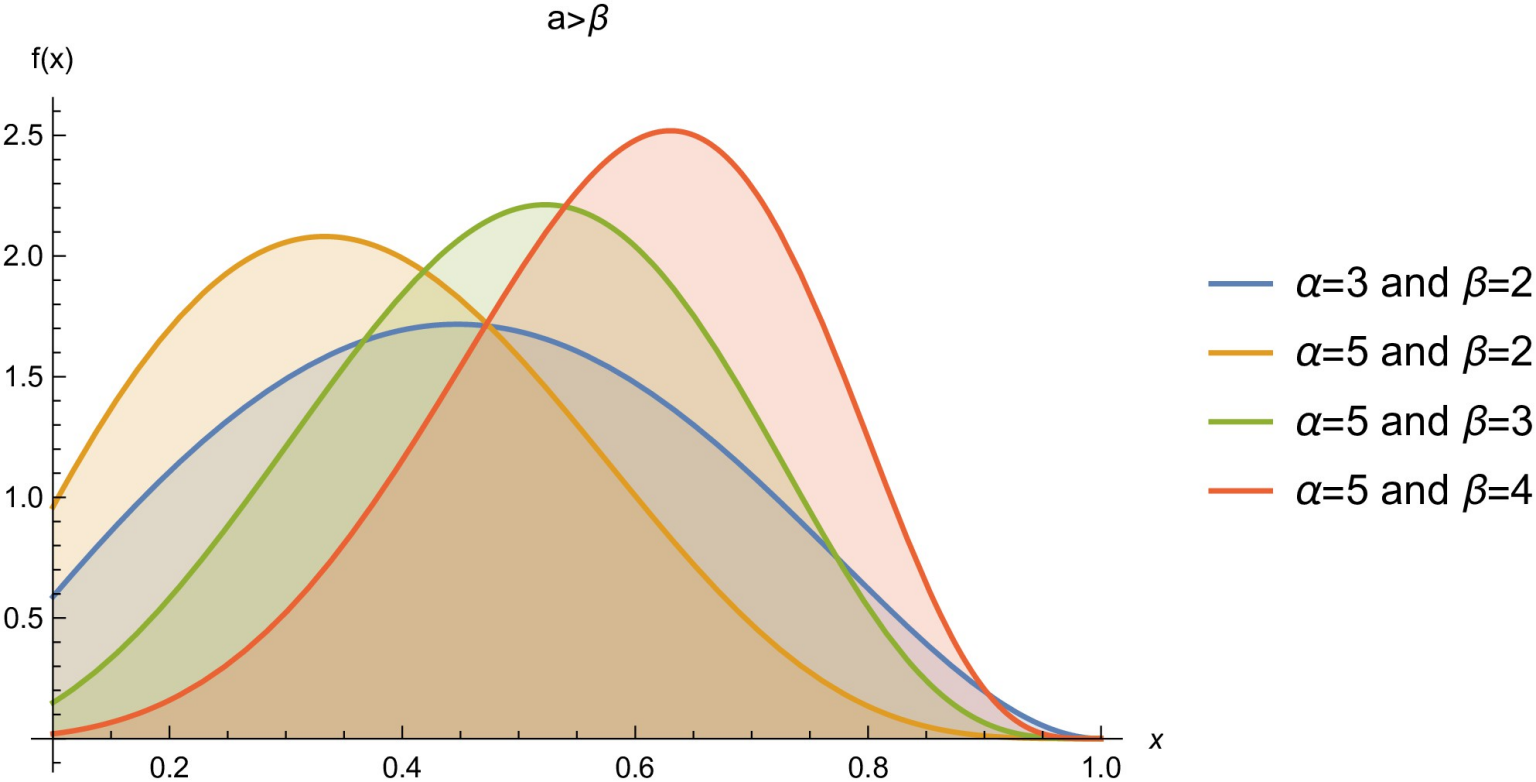
pdf of *Kum*(*α*, *β*), when *α* > *β*.

**Fig 4:**
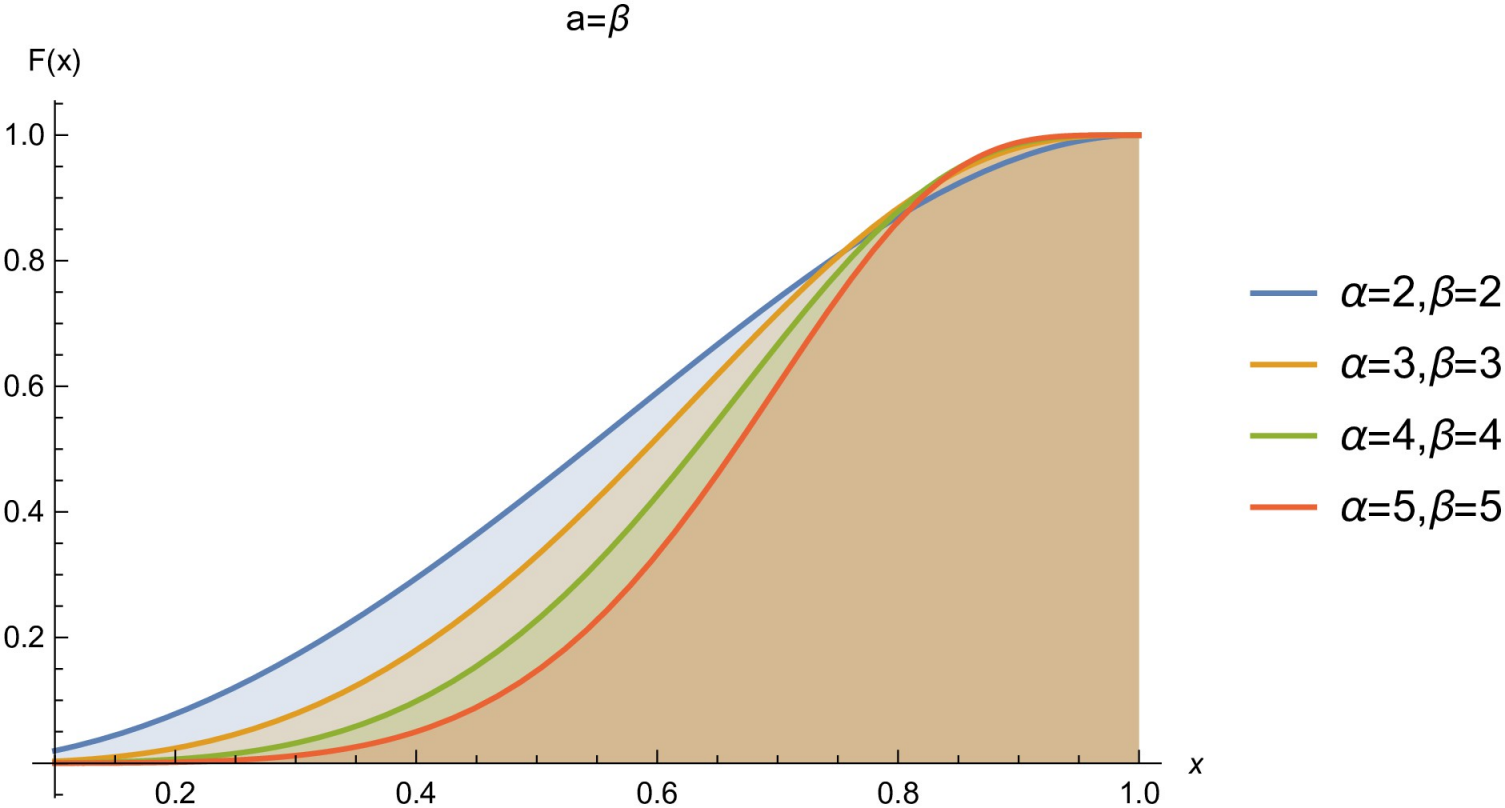
cdf of *Kum*(*α*, *β*), when *α* = *β*.

**Fig 5:**
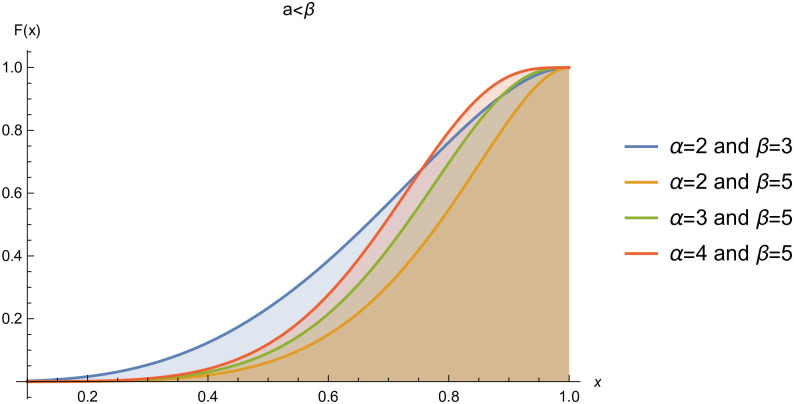
cdf of *Kum*(*α*, *β*), when *α* < *β*.

**Fig 6:**
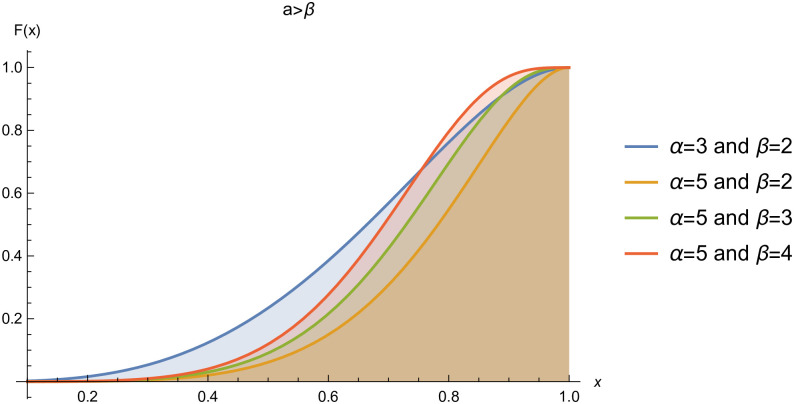
cdf of *Kum*(*α*, *β*), when *α* > *β*.

This distribution was developed by [16]. [16, 17] have shown that Kumaraswamy’s distribution can be used to approximate many different distributions depending on the parameters *α* and *β*, for example, uniform, triangular, triangular, and many others. The Kumaraswamy distribution has received considerable attention in the literature among others [18–23]. The probability density function (*pdf*) and the cumulative distribution function (*cdf*) of a two-parameter Kumaraswamy random variable *X* is written as

$$\begin{array}{ccc} f(x) & = & \alpha \beta x^{\beta -1}{\left(1-x^{\beta }\right)}^{\alpha -1},\;0<x<1; \end{array}$$ (1)

$$\begin{array}{ccc} F(x) & = & 1-{\left(1-x^{\beta }\right)}^{\alpha }, \end{array}$$ (2)

respectively, where *α* > 0 and *β* > 0 are the shape parameters. For simplicity we will use *Kum*(*α*, *β*) to represent the two-parameter Kumaraswamy probability density function. The plots of the *pdf* and the *cdf* of the *Kum*(*α*, *β*) distribution for selected parameter values are represented in Figs (1)–(6).

The objective of this paper is to develop a new competitive methods of parameter estimation for the Kumaraswamy distribution and to identify the most efficient estimator. These methods include the probability weighted moment, the self-determined probability weighted-moment and the principle of maximum entropy.

The performance of each of these methods is studied and compared with their counter traditional methods, such as the maximum likelihood estimation and the method of moment estimators using extensive simulations. The estimates were compared in terms of their resulting biases, mean squared error, coverage probability and length of their confidence intervals. The construction of the confidence intervals of the unknown parameters is obtained using bootstrap method (see [24]). In addition, the bootstrap method is also used to calculate the bootstrap bias, standard error, lower and upper confidence interval limits for the real life data sets.

## Methods of estimation

In this section, we discuss the principle of maximum entropy estimators (*POME*) in details, followed by the method of moments estimation (*MOM*) and its variants such as the probability weighted moments and the self-determined probability weighted moments and finally the maximum likelihood estimation method, each followed by the procedure adopted for the estimation.

### Principle of maximum entropy (*POME*)

Among the parameter estimation methods, entropy, which is a measure of uncertainty of random variables, has attracted much attention and has been used for a variety of applications in hydrology [25].

The Shannon entropy function *H*(*f*) for a continuous random variable *X* can be expressed as:

$$\begin{array}{c} H\left(f\right)=-\int_{-\infty }^{\infty }f\left(x;\theta \right)\;\text{ln}\;f\left(x;\theta \right)dx\;\;\;\;\;\;\text{with}\;\;\int_{-\infty }^{\infty }f\left(x;\theta \right)dx=1. \end{array}$$ (3)

To estimate the parameters *α* and *β* of Eq (1), the *POME* method needs to maximize *H*(*f*) by establishing a relationship between the constraints and the Lagrange multipliers. This can be achieved by, specifying the suitable constraints, deriving the entropy function of the *Kum*(*α*, *β*) distribution, and finally, concluding the relationship between the Lagrange multipliers and these constraints. A complete mathematical discussion of this method can be found in [13], Levine and [14], Sigh and [15, 26, 27].

#### Specification of constraints

Since the maximization of the entropy relays on the constraints to be satisfied by *Kum*(*α*, *β*) function, the first step in the application of the *POME* is to determine its constraints. We do so, by taking the natural logarithm of Eq (1) which is written as

$$\begin{array}{c} \text{ln}\;f\left(x\right)=\;\text{ln}\;(\alpha \beta )+(\beta -1)\;\text{ln}\;(x)-(\alpha -1)\;\text{ln}\;(1-x^{\beta }). \end{array}$$ (4)

Multiplying Eq (4) by [−*f*(*x*)] and integrating from 0 to 1, we obtain the entropy function:

$$\begin{array}{c} H\left(f\right)=-\overset{}{\int_{0}^{1}f\left(x\right)[\text{ln}\;(\alpha \beta )+(\beta -1)\;\text{ln}\;(x)-(\alpha -1)\;\text{ln}\;(1-x^{\beta })]}dx. \end{array}$$ (5)

To maximize *H*(*f*) in Eq (5), the following constraints should be satisfied.

$$\begin{array}{ccc} C1 & : & \;\int_{0}^{1}f\left(x;\theta \right)dx=1, \end{array}$$ (6)

$$\begin{array}{ccc} C2 & : & \;\int_{0}^{1}\;\text{ln}\;(x)f\left(x;\theta \right)dx=E\;\text{ln}\;(X), \end{array}$$ (7)

$$\begin{array}{ccc} C3 & : & \;\int_{0}^{1}\;\text{ln}\;(1-x^{\beta })f\left(x;\theta \right)dx=E\;\text{ln}\;(1-X^{\beta }), \end{array}$$ (8)

where, *E*(*) denotes the expectation of a bracketed quantity, *C*_1_, *C*_2_ and *C*_3_, are unique constraints (see [15]). To derive the *POME* method for the estimation of the parameters of *Kum*(*α*, *β*), Eqs (6)–(8) become the constraints. A complete mathematical discussion of the rational for deriving the constraints in this manner can be found in [13, 14, 15].

#### Construction of the zeroth Lagrange multiplier

The least biased pdf, *f*(*x*), consistent with Eqs (6)–(8), and corresponding to the *POME* takes the following form

$$\begin{array}{c} f\left(x\right)=e^{-\lambda_{0}-\lambda_{1}\;\text{ln}\;(x)-\lambda_{2}\;\text{ln}\;(1-x^{\beta })}, \end{array}$$ (9)

where λ_0_, λ_1_ and λ_2_ are the Lagrange multipliers. The λ*s* represent the information content of each constraint i.e. if λ_*i*_ = 0 this means that the *i*th constraint is redundant and has no informational value. Hence, It doesn’t reduce the level of uncertainty. The mathematical rational for Eq (9) has been presented in [14].

By applying Eq (9) to the total probability condition in Eq (3), one obtains:

$$\begin{array}{ccc} e^{\lambda_{0}} & = & \int_{0}^{1}x^{-\lambda_{1}}{\left(1-x^{\beta }\right)}^{-\lambda_{2}}dx\\ & = & \frac{\Gamma (1-\lambda_{2})\Gamma (\frac{1-\lambda_{1}}{\beta })}{\beta \;\Gamma (1-\lambda_{2}+\frac{1-\lambda_{1}}{\beta })}, \end{array}$$ (10)

Taking the logarithm of Eq (10) results in the zeroth Lagrange λ_0_ multiplier as a function of Lagrange multiplier λ_1_ and λ_2_, with expression given as:

$$\begin{array}{c} \lambda_{0}=\;\text{ln}\;\Gamma (1-\lambda_{2})+\;\text{ln}\;\Gamma (\frac{1-\lambda_{1}}{\beta })-\;\text{ln}\;\beta -\;\text{ln}\;\Gamma (1-\lambda_{2}+\frac{1-\lambda_{1}}{\beta }). \end{array}$$ (11)

The inverse of Eq (10) is:

$$\begin{array}{c} e^{-\lambda_{0}}=\frac{\beta \;\Gamma (1-\lambda_{2}+\frac{1-\lambda_{1}}{\beta })}{\Gamma (1-\lambda_{2})\Gamma (\frac{1-\lambda_{1}}{\beta })}. \end{array}$$ (12)

#### Derivation of entropy function

Introduction of Eq (12) in Eq (9) produces

$$\begin{array}{c} f\left(x\right)=\frac{\beta \Gamma (1-\lambda_{2}+\frac{1-\lambda_{1}}{\beta })}{\Gamma (1-\lambda_{2})\Gamma (\frac{1-\lambda_{1}}{\beta })}\;\text{ln}\;{\left(x\right)}^{-\lambda_{1}}\;\text{ln}\;{\left(1-x^{\beta }\right)}^{-\lambda_{2}}. \end{array}$$ (13)

By contrasting Eq (13) with Eq (1) we recognize that

$$\begin{array}{c} \lambda_{1}=1-\beta \;\&\;\lambda_{2}=1-\alpha . \end{array}$$ (14)

Using Eq (13), the entropy function, given by Eq (3), can be written as

$$\begin{array}{c} H\left(f\right)=-\;\text{ln}\;(\frac{\beta \Gamma (1-\lambda_{2}+\frac{1-\lambda_{1}}{\beta })}{\Gamma (1-\lambda_{2})\Gamma (\frac{1-\lambda_{1}}{\beta })})+\lambda_{1}E\;\text{ln}\;(X)+\lambda_{2}E\;\text{ln}\;(1-X^{\beta }). \end{array}$$ (15)

#### Relation between the distribution parameters and the constraints

According to [15], the relation between the distribution parameters and the constraints are obtained by taking partial derivatives of Eq (15) with respect to the Lagrange multipliers as well as the distribution parameters, and then equating (each of) these derivative to zero, and making use of the constraints. To that end, taking partial derivatives of Eq (15) with respect to λ_1_, λ_2_ and *β* separately and equating each derivative to zero yields:

$$\begin{array}{ccc} \frac{\partial H}{\partial \lambda_{1}} & = & \frac{\psi (\frac{1-\lambda_{1}}{\beta })-\psi (\frac{1-\lambda_{1}}{\beta }+1-\lambda_{2})}{\beta }-E\;\text{ln}\;X=0, \end{array}$$ (16)

$$\begin{array}{ccc} \frac{\partial H}{\partial \lambda_{2}} & = & \psi (1-\lambda_{2})-\psi (\frac{1-\lambda_{1}}{\beta }+1-\lambda_{2})-E\;\text{ln}\;(1-X^{\beta })=0, \end{array}$$ (17)

$$\begin{array}{ccc} \frac{\partial H}{\partial \beta } & = & \frac{\beta +(1-\lambda_{1})[\psi (\frac{1-\lambda_{1}}{\beta })-\psi (\frac{1-\lambda_{1}}{\beta }+1-\lambda_{2})]}{\beta^{2}}+\lambda_{2}E(\frac{X^{\beta }\;\text{ln}\;X}{1-X^{\beta }})=0, \end{array}$$ (18)

where $\psi \left(t\right)=\frac{d}{dt}\text{ln}\Gamma \left(t\right)$ is the digamma function. Introduction of Eq (14) into Eqs (16)–(18) and recalling Eqs (16)–(18) yields respectively:

$$\begin{array}{c} \frac{\psi (\alpha +1)-\psi (1)}{\beta }+E\;\text{ln}\;\left(X\right)=0, \end{array}$$ (19)

$$\begin{array}{c} \psi (\alpha +1)-\psi (\alpha )+E\;\text{ln}\;(1-X^{\beta })=0, \end{array}$$ (20)

$$\begin{array}{c} \frac{1+\psi (1)-\psi (\alpha +1)}{\beta }+(1-\alpha )E(\frac{X^{\beta }\;\text{ln}\;X}{1-X^{\beta }})=0 \end{array}$$ (21)

Clearly, Eq (21) doesn’t hold. Therefore, the parameter estimation equations for *POME* consist of Eqs 19 and 20. The expectations of Eqs 19 and 20 are replaced by their sample estimates, and the simplifications of 19 and 20 leads to

$$\begin{array}{c} \frac{\psi (\alpha +1)-\psi (1)}{\beta }+\overset{n}{\sum_{i=1}}\frac{\;\text{ln}\;(x_{i})}{n}=0 \end{array}$$ (22)

$$\begin{array}{c} \psi (\alpha +1)-\psi (\alpha )+\overset{n}{\sum_{i=1}}\frac{\;\text{ln}\;(1-x_{i}^{\beta })}{n}=0 \end{array}$$ (23)

### Method of moments(*MOM*)

The method of moments, estimating the parameters of the probability distribution by matching the sample moments

$$\begin{array}{ccc} m_{1} & = & \frac{1}{n}\underset{i=1}{\sum^{n}}x_{i}=\bar{x}, \end{array}$$ (24)

$$\begin{array}{ccc} m_{2} & = & \frac{1}{n}\underset{i=1}{\sum^{n}}x_{i}^{2} \end{array}$$ (25)

with the theoretical moments using Eqs 26 and 27 to obtain the *MOM* estimates (${\hat{\alpha }}_{mom}$ and ${\hat{\beta }}_{mom}).$

$$\begin{array}{ccc} EX & = & \frac{\Gamma (\frac{1}{\beta }+1)\Gamma (1+\alpha )}{\Gamma (1+\alpha +\frac{1}{\beta })}, \end{array}$$ (26)

$$\begin{array}{ccc} EX^{2} & = & \frac{\alpha \Gamma (\alpha )\Gamma (\frac{2}{\beta }+1)}{\Gamma (1+\alpha +\frac{2}{\beta })}. \end{array}$$ (27)

### Probability weighted moments(*PWM*)

The probability weighted moments of a random variable *X* with cumulative distribution function *F*(*x*) = *P*(*X* ≤ *x*) and quantile *X* = *X*(*F*) are formally defined as:

$$\begin{array}{ccc} M_{p,r,s} & = & E[X^{p}F^{r}{\left(1-F\right)}^{s}]\\ & = & \int_{0}^{1}{\left[X(F)\right]}^{p}F^{r}{\left(1-F\right)}^{s}dF \end{array}$$ (28)

where *M*_*p*,*r*,*s*_ is the probability weighted moment of order (*p*, *r*, *s*), *E* is the expectation operator and *p*, *r*, and *s* are real numbers. If *r* = *s* = 0 and *p* is a nonnegative integer, then *M*_*p*,0,0_ represents the conventional moment about the origin of order *p*. Two useful sets of *M*_*p*,*r*,*s*_ are defined as:

$$\begin{array}{c} \alpha_{s}\equiv M_{1,0,s}=\int_{0}^{1}X\left(F\right){\left(1-F\right)}^{s}dF;\;s=0,1,2,\ldots \end{array}$$ (29)

and

$$\begin{array}{c} \beta_{r}\equiv M_{1,r,0}=\int_{0}^{1}X\left(F\right)F^{r}dF;\;r=0,1,2,\ldots \end{array}$$ (30)

The two *PWM* sets *α*_*s*_&*β*_*r*_, are linear combinations of each other. [4] favored *α*_*s*_, [6] favored *β*_*r*_. Therefore, any one of them can be used, whichever is possible. In practice, one chooses the moments for which Eq (2) can be most easily solved analytically.

For nonnegative integers *s* & *r*, [5] introduced unbiased estimators of *α*_*s*_ and *β*_*r*_, which are based on the ordered sample *x*_(1)_, *x*_(2)_, …, *x*_(*n*)_ from the distribution *F*. Which are defined as:

$$\begin{array}{c} {\hat{\alpha }}_{s}=\frac{1}{n}\underset{i=1}{\sum^{n}}x_{\left(i\right)}\frac{\left(\begin{array}{c} n-i\\ s \end{array}\right)}{\left(\begin{array}{c} n-1\\ s \end{array}\right)}, \end{array}$$ (31)

and

$$\begin{array}{c} {\hat{\beta }}_{r}=\frac{1}{n}\underset{i=1}{\sum^{n}}x_{\left(i\right)}\frac{\left(\begin{array}{c} i-1\\ r \end{array}\right)}{\left(\begin{array}{c} n-1\\ r \end{array}\right)}, \end{array}$$ (32)

where

$$\begin{array}{c} \frac{\left(\begin{array}{c} n-i\\ s \end{array}\right)}{\left(\begin{array}{c} n-1\\ s \end{array}\right)}, \end{array}$$ (33)

and

$$\begin{array}{c} \frac{\left(\begin{array}{c} i-1\\ r \end{array}\right)}{\left(\begin{array}{c} n-1\\ r \end{array}\right)}, \end{array}$$ (34)

are estimates of the exceedance (1 − *F*(*x*)) and the non-exceedance *F*(*x*) probabilities, respectively. These estimates are not based on the assumed distribution, but are based solely on the position of *x*_(*i*)_ within the ordered sample.

For the *Kum*(*α*, *β*) distribution, we prefer to work with the *PWM* of the form *α*_*s*_ which is given by Eq (29). The inverse function of Eq (1) is given by

$$\begin{array}{c} X(F)={\left[1-{\left(1-F\right)}^{\frac{1}{\alpha }}\right]}^{\frac{1}{\beta }} \end{array}$$ (35)

The *PWMs* (*α*_*s*_) for *Kum*(*α*, *β*) is given by:

$$\begin{array}{ccc} \alpha_{s} & = & \int_{0}^{1}{\left[1-{\left(1-F\right)}^{\frac{1}{\alpha }}\right]}^{\frac{1}{\beta }}{\left[1-F\right]}^{s}dF\\ & = & \frac{\pi \alpha Csc(\frac{\pi }{\beta })\Gamma (\alpha (s+1))}{\beta \;\Gamma (1+(s+1)\alpha +\frac{1}{\beta })\Gamma (1-\frac{1}{\beta })};\;\;\;\beta >1, \end{array}$$ (36)

where *Csc*(*) is the cosecant function of the bracketed quantity. Since the Kumaraswamy distribution is a two-parameter distribution, then only the first two *PWM*; *α*_0_ and *α*_1_ are needed. They are given as follows:

$$\begin{array}{ccc} \alpha_{0} & = & \frac{\pi \;\alpha \;\Gamma (\alpha )Csc(\frac{\pi }{\beta })}{\beta \;\Gamma (1+\alpha +\frac{1}{\beta })\Gamma (1-\frac{1}{\beta })}; \end{array}$$ (37)

$$\begin{array}{ccc} \alpha_{1} & = & \frac{\pi \;\alpha \;\Gamma (2\alpha )Csc(\frac{\pi }{\beta })}{\beta \;\Gamma (1+2\alpha +\frac{1}{\beta })\Gamma (1-\frac{1}{\beta })}; \end{array}$$ (38)

To obtain the *PWM* estimates ${\hat{\alpha }}_{PWM}$ and ${\hat{\beta }}_{PWM},$ the population *PWMs*: *α*_0_;*α*_1_ in Eqs (37) & (38) are replaced by their sample estimators $\left({\hat{\alpha }}_{0},{\hat{\alpha }}_{1}\right)$ using (31), the resulting equations are:

$$\begin{array}{ccc} {\hat{\alpha }}_{0} & = & \frac{1}{n}\underset{i=1}{\sum^{n}}x_{\left(i\right)}=\bar{x} \end{array}$$ (39)

$$\begin{array}{ccc} {\hat{\alpha }}_{1} & = & \frac{1}{n(n-1)}\underset{i=1}{\sum^{n}}(n-i)x_{\left(i\right)}. \end{array}$$ (40)

Note that based on the ordered sample *x*_(1)_, *x*_(2)_, …, *x*_(*n*)_ of size *n* from the distribution *F*, ${\hat{\alpha }}_{s}$ in Eq (31) is an unbiased estimator of *α*_*s*_ [5, 6].

The value of the Kumaraswamy parameters can be determined by substituting the estimators given by Eqs (39)&(40) in the Eqs (37)&(38).

### Self-determined probability weighted-moments(*SD*-*PWM*)

In order to accurately estimate the distribution parameters, we presumed that the sample observations follow the *Kum*(*α*, *β*) distribution, thus conduct some relevant behavior of the distribution. An inspection of Eq (31) shows that the exceedance probability is not assigned to *x*_*i*_ according to the assumed distribution, but rather based solely on the position of *x*_*i*_ within the ordered sample. As mentioned before in the introduction section, the method *SD*-*PWM* was developed as an effort to improve estimation performance of the *PWM* method by using the given distribution. The method of *SD*-*PWM* assumes that the exceedance probability of the observations can be assigned via the cumulative distribution function of the assumed distribution, therefore, the *SD*-*PWM* sample estimators ${\tilde{\alpha }}_{s}$ for *α*_*s*_ is defined as:

$$\begin{array}{ccc} {\tilde{\alpha }}_{s} & = & \frac{1}{n}\underset{i=1}{\sum^{n}}{\left[1-F(x_{\left(i\right)})\right]}^{s}x_{\left(i\right)};\;s=0,1,2,\ldots \end{array}$$ (41)

$$\begin{array}{ccc} & = & \frac{1}{n}\underset{i=1}{\sum^{n}}{\left[{\left(1-x_{\left(i\right)}^{\beta }\right)}^{\alpha }\right]}^{s}x_{\left(i\right)}. \end{array}$$ (42)

Since the Kumaraswamy distribution is a two-parameter distribution, then only the first two *SD*-*PWM* samples are needed, hence,

$$\begin{array}{ccc} {\tilde{\alpha }}_{0} & = & \bar{x}, \end{array}$$ (43)

$$\begin{array}{ccc} {\tilde{\alpha }}_{1} & = & \frac{1}{n}\underset{i=1}{\sum^{n}}{\left(1-x_{\left(i\right)}^{\beta }\right)}^{\alpha }x_{\left(i\right)}. \end{array}$$ (44)

The value of the Kumaraswamy parameters using the method of *SD*-*PWM* can be obtained by replacing *α*_0_ and *α*_1_ in Eqs (37) and (38) by ${\tilde{\alpha }}_{0}$ and ${\tilde{\alpha }}_{1}$ in Eqs (43) and (44).

### Maximum likelihood method

Let *x*_1_, *x*_2_, …, *x*_*n*_ be a random sample from *Kum*(*α*, *β*), using Eq (1) the log-likelihood function is given by

$$\begin{array}{c} LogL=n\;\text{log}(\alpha \beta )+(\beta -1)\underset{i=1}{\sum^{n}}\text{log}(x_{i})+(\alpha -1)\underset{i=1}{\sum^{n}}\text{log}(1-x_{i}^{\beta }). \end{array}$$ (45)

The *MLEs* of the parameters *α* and *β*, denoted by ${\tilde{\alpha }}_{MLE}$ and ${\hat{\beta }}_{MLE}$ respectively, can be obtained by taking the first derivative of (45) with respect to *α* and *β* and then equating the normal equations to 0 as follows:

$$\begin{array}{ccc} \frac{\partial LogL}{\partial \alpha } & = & \frac{n}{\alpha }-\underset{i=1}{\sum^{n}}\text{log}(1-x_{i}^{\beta })=0, \end{array}$$ (46)

$$\begin{array}{ccc} \frac{\partial LogL}{\partial \beta } & = & \frac{n}{\beta }+\underset{i=1}{\sum^{n}}\text{log}(x_{i})-(\alpha -1)\underset{i=1}{\sum^{n}}\frac{x_{i}^{\beta }\;\text{log}x_{i}}{1-x_{i}^{\beta }}=0. \end{array}$$ (47)

Note that there is no explicit solutions to Eqs (46) and (47). Hence, numerical methods like Newton–Raphson method can be used to obtain *MLEs* of *α* and *β*.

## Real life data

This section considers two real-life data sets, namely maximum flood level and relief time for 50 arthritic patients, to demonstrate the proposed method and verify its effectiveness. The validity of the Kumaraswamy model was checked using Kolmogorov-Smirnov (*K* − *S*), Anderson-Darling (*A* − *D*), and chi-square tests.

### Example 1

The data for this application were obtained in a civil engineering context. It represents the maximum flood level (in millions of cubic feet per second) for the Susquehanna River at Harrisburg, Pennsylvania. The numbers in this data represent the maximum flood level for four years, the first number being 0.654 for the period 1890–1893, and the last one being 0.265, which is for the time period 1966–1969. The data were utilized by [28] and it is given in Table (1).

**Table 1:** The maximum flood level (in million of *feet*^3^/second) for the Susquehanna River at Harrisburg, Pennsylvania.

0.654	0.613	0.315	0.449	0.297	0.402	0.379	0.423	0.379	0.324
0.269	0.740	0.418	0.412	0.494	0.416	0.338	0.392	0.484	0.265

**Fig 7:**
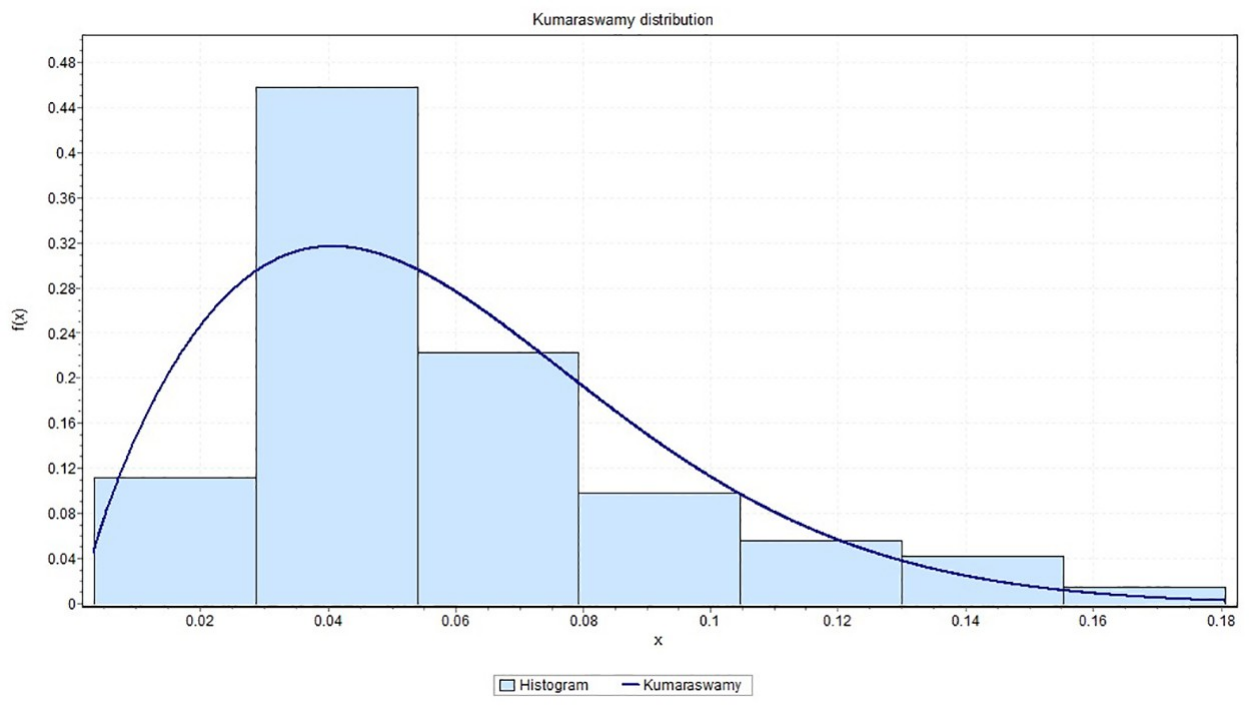
The histogram of the data set and its fitted density function to the maximum flood level data in million of *feet*^3^/second.

**Fig 8:**
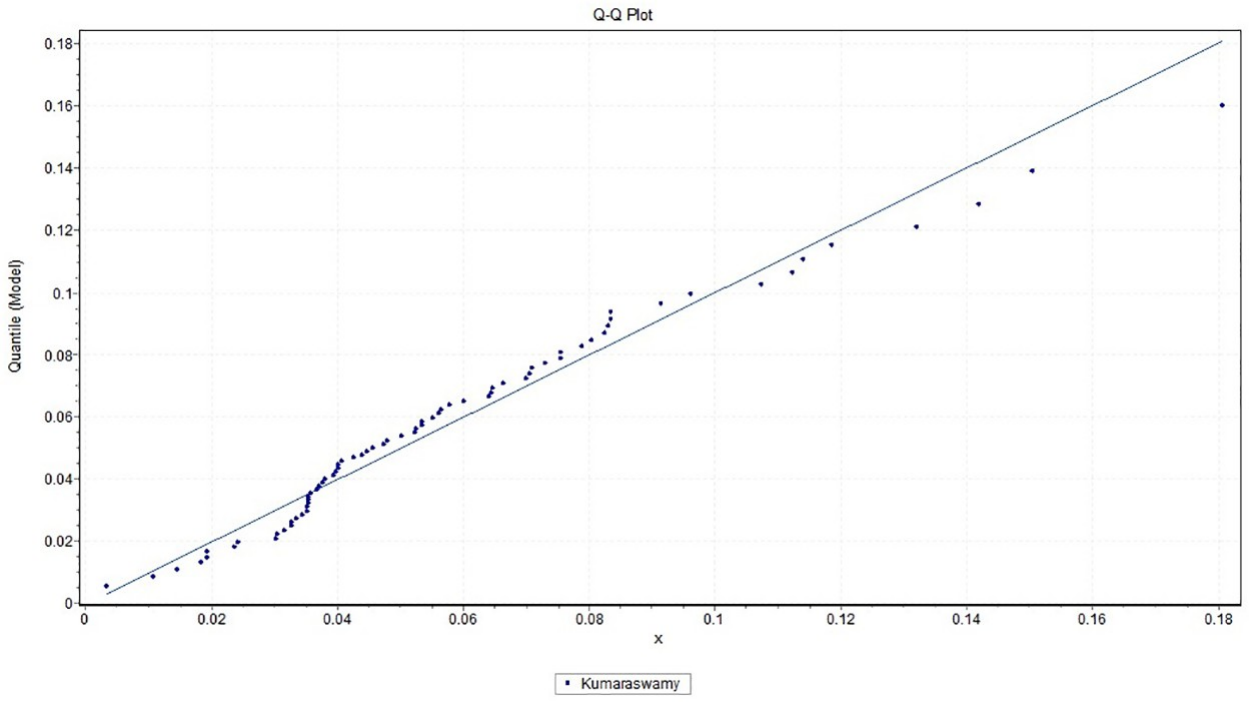
Plot of the empirical quantile of Kumaraswamy distribution fitted to the maximum flood level data in million of *feet*^3^/second.

It is observed that *K*-*S* = 0.21091 with *p*_*value*_ = 0.29281, *A*-*D* = 0.93218 and chi-square distance = 2.4424 with a corresponding *p*_*value*_ = 0.29488. This indicates that the Kumaraswamy model provides a good fit to the above data. Fig (7) gives the histogram of the data-set and the plots of the fitted density. The QQ plot in Fig (8) suggests that Kumaraswamy is very suitable for this data. In contrast, some hydrologist believe flooding can be assessed by unbounded distributions (see [29]).

Parametric bootstrap percentile method is used to compute the parametric estimates, the bootstrap estimates (BootEst) and their corresponding standard error (StdErr). A 95% confidence interval is calculated and reported in terms of (LCL, UCL). The output of the bootstrap analysis along with the parameter estimates are summarized in Table (2).

**Table 2:** Parameter estimates, standard error, length and coverage of a 95% confidence interval based on bootstrap re-samples from the maximum flood level data in million of *feet*^3^/second.

	*α*					*β*				
Method	Parameter estimates	BootEst	SthErr	LCL	UCL	Parameter estimates	BootEst	SthErr	LCL	UCL
*MLE*	11.7892	10.6103	1.9717	5.9395	11.4083	3.3632	3.2182	0.2418	2.7404	3.7185
*MOM*	15.0000	11.2644	1.5628	6.4408	12.5506	3.6263	3.2484	0.2571	2.7588	3.7876
*PWM*	11.7896	11.3297	0.6174	11.8764	12.0000	3.3673	3.3188	0.2337	2.8624	3.8137
*SDPWM*	12.2114	11.9161	1.5292	6.3078	12.0018	3.3083	3.3065	0.2686	2.8355	3.8669
*POME*	13.7749	12.2377	2.4927	6.3742	14.1111	3.6045	3.4561	0.2608	2.9006	3.9606

### Example 2

Our second data set were taken from a clinical trial aimed at testing the efficacy of an analgesic. In Table (3), relief times (in hours) are shown for 50 arthritic patients treated with a fixed dosage of this medication. These data were first utilized by [30] and later by [31].

**Table 3:** Relief time (in hours) for 50 arthritic patients.

0.70	0.84	0.58	0.50	0.55	0.82	0.59	0.71	0.72	0.61
0.62	0.49	0.54	0.36	0.36	0.71	0.35	0.64	0.84	0.55
0.59	0.29	0.75	0.46	0.46	0.60	0.60	0.36	0.52	0.68
0.80	0.55	0.84	0.34	0.34	0.70	0.49	0.56	0.71	0.61
0.57	0.73	0.75	0.44	0.44	0.81	0.80	0.87	0.29	0.50

**Fig 9:**
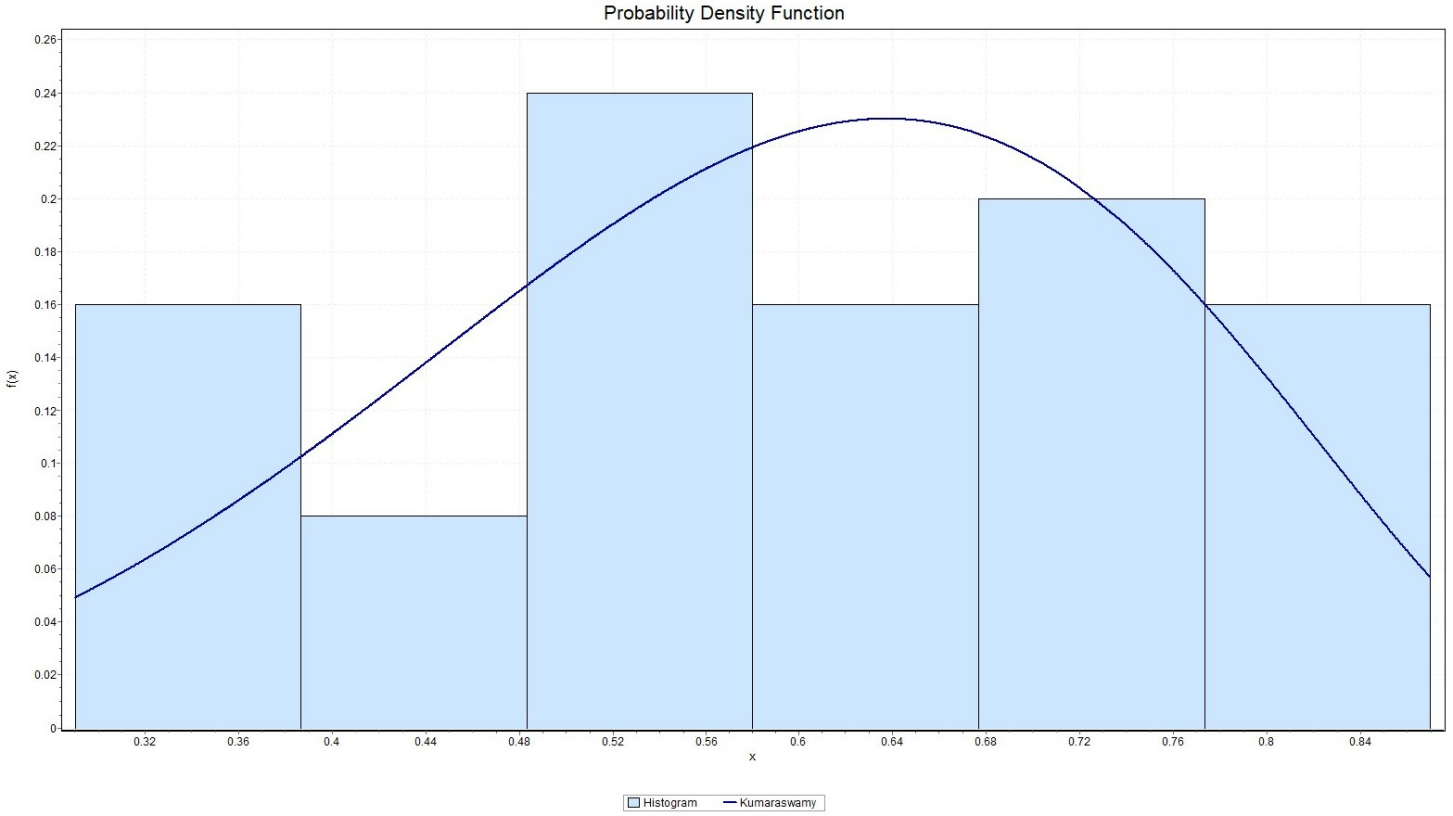
The histogram of the data set and its fitted density function of the patient relief times.

**Fig 10:**
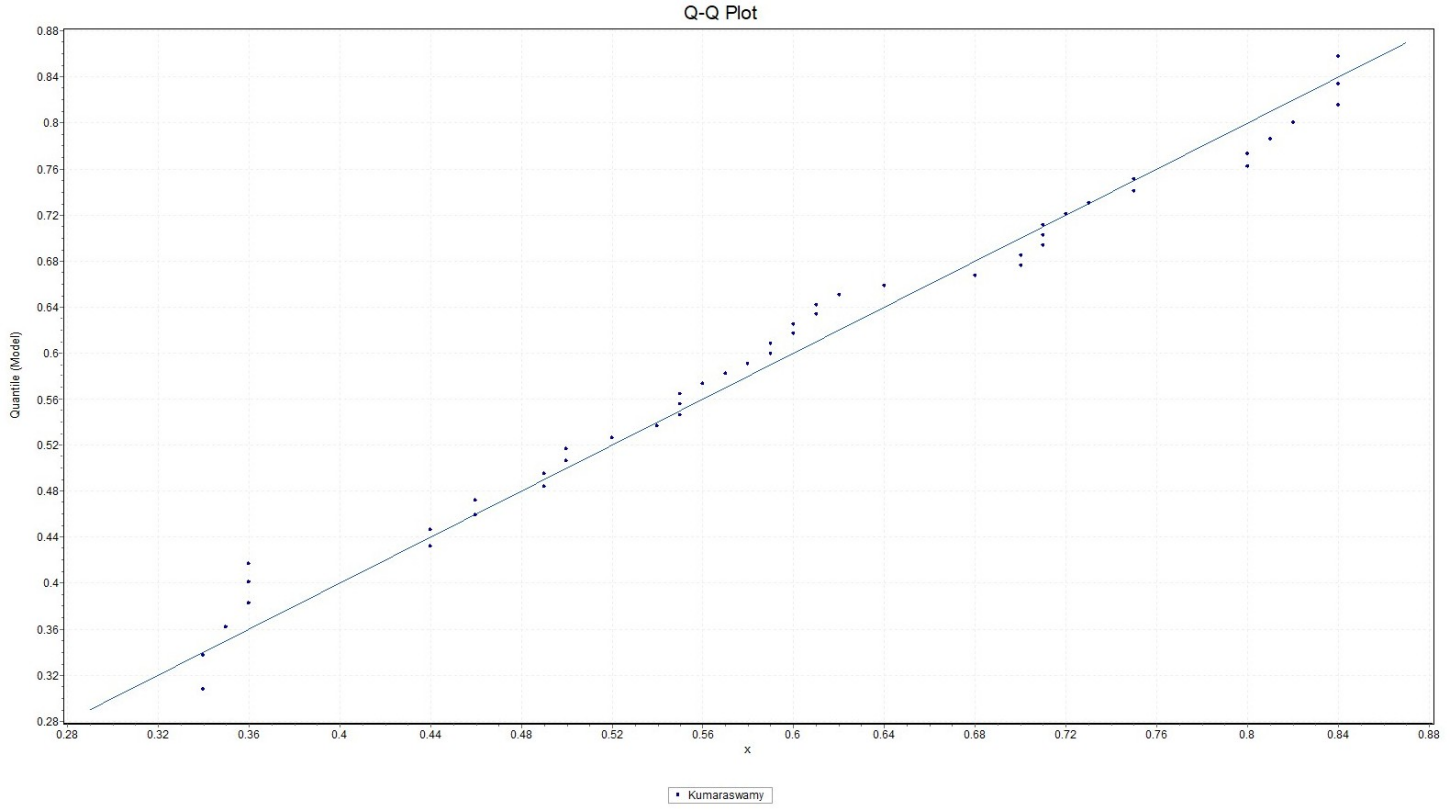
Plot of the empirical quantile of Kumaraswamy distribution fitted to the patient relief times.

The legitimacy of the Kumaraswamy model was checked. It is observed that *K*-*S* = 0.08578 with *p*_*value*_ = 0.8249, *A*-*D* = 0.39141 and chi-square distance = 2.3616 with a corresponding *p*_*value*_ = 0.66958. This indicates that the Kumaraswamy model provides a good fit to the above data. Fig (9) gives the histogram of the data-set and the plot of the fitted density. The QQ plot in Fig (10) suggests that Kumaraswamy is very suitable for the precipitation data.

Parametric bootstrap percentile method is used to compute the parameter estimates, the bootstrap estimates (BootEst) and their corresponding standard error (StdErr). A 95% confidence interval is calculated and reported in terms of (LCL, UCL). The output of the bootstrap analysis along with the parameter estimates are summarized in Table (4). Tables (2) and (4) reveal that PWM-based estimates regularly outperform all other estimating strategies. Their *Bias* and standard error are the least, and their confidence interval is the shortest.

**Table 4:** Parameter estimates, standard error, length and coverage of a 95% confidence interval based on bootstrap resamples from the patient relief times.

	*α*					*β*				
Method	Parameter estimates	BootEst	SthErr	LCL	UCL	Parameter estimates	BootEst	SthErr	LCL	UCL
*MLE*	4.2513	4.5248	0.9736	3.0794	6.4930	3.7562	3.8379	0.4059	3.1274	4.7124
*MOM*	4.2984	4.5937	1.2533	2.8728	7.3888	3.7594	3.8341	0.4447	3.0370	4.8096
*PWM*	4.3006	4.0168	0.6700	2.7428	5.0000	3.7529	3.6132	0.2997	2.9394	4.0000
*SDPWM*	4.3697	4.5148	1.0828	2.6033	6.0000	3.7890	3.7975	0.4267	2.9744	4.6350
*POME*	4.3057	4.5728	1.0301	3.1099	6.6605	3.7886	3.8684	0.4053	3.1484	4.7687

## Simulation study

In this section, a simulation study is conducted to compare the performance of the different estimation procedures discussed in the previous sections. The performance of the different estimators is compared in terms of *Bias* and mean squared error (*MSE*). Suppose ${\hat{\theta }}_{i}(=\alpha_{i},\beta_{i})$ is the estimate of *θ* (= *α*, *β*) for the *i*-*th* simulated data set, then the absolute Bias (*Bias*) and the *MSE* are computed as follows: $Bias=\frac{1}{1000}\sum_{i=1}^{1000}\left|{\hat{\theta }}_{i}-\theta \right|$ & $MSE=\frac{1}{1000}\sum_{i=1}^{1000}({\hat{\theta }}_{i}-\theta {)}^{2}$.

We perform the simulation study using SAS/IML. We replicate the process 1000 times. In each replication, a random sample of size *n* (= 20, 30,50, 80,100, 200, 300, 500, 1000) is drawn from the Kumaraswamy distribution. The true parameter values used in the data generating process are (*α*, *β*) = (2,2), (2,3), (2,4), (2,5), (2,10), (3,2), (3,3), (3,4), (3,5), (3,10), (4,2), (4,3), (4,4), (4,5), (4,10), (5,2), (5,3), (5,4), (5,5), (5,10). In each case, we compute 95% symmetric percentile bootstrap confidence interval based on 500 bootstrap samples. We repeat the process 1000 times and obtain the average lengths (*L*) of the confidence intervals and their coverage percentages (*CP*). All results are reported in Tables 5–10.

- It is immediate from Tables 5–7 that as the sample size increases, all estimators demonstrate the property of consistency, meaning their *MSE* values approach zero.
- The study also depicts that the estimates based on *PWM* outperforms all other estimates in terms of *Bias* and *MSE* values, demonstrating that this technique is useful in estimating the shape parameters of the Kumaraswamy distribution.
- It should also be noted that the estimates based on *POME* are equivalent to the estimates based on *MLE* in terms of *Bias* and *MSE* values, and this is true for all values of *n*, *α* and *β*.
- For all estimating approaches, the *Bias* of both $\hat{\alpha }$ and $\hat{\beta }$ decreases as *n* increases, as expected.
- In terms of *Bias* and *MSE* values, Tables 5–7 clearly illustrate that *POME*-based estimates and *MlE*-based estimates are nearly identical.
- Overall, the simulation results suggest that with a large sample size *n*, the differences in *Bias* and *MSE* between *MLE*, *MOM*, *SD*-*PWM*, and *POME* methods of estimation become minimal.

**Table 5:** *Bias* and *MSE* (parentheses) for the estimators of *α* and *β* when *α* = *β* and for different choices of *n*.

		*α* = *β* = 2		*α* = *β* = 3		*α* = *β* = 4		*α* = *β* = 5	
		$\hat{\alpha }$	$\hat{\beta }$	$\hat{\alpha }$	$\hat{\beta }$	$\hat{\alpha }$	$\hat{\beta }$	$\hat{\alpha }$	$\hat{\beta }$
n = 20	*MLE*	0.4962(0.0752)	0.2224(0.0213)	0.8394(0.1914)	0.2959(0.0384)	1.0929(0.3027)	0.3415(0.0543)	1.1873(0.3420)	0.3453(0.0660)
	*MOM*	0.4596(0.0737)	0.1943(0.0212)	0.8258(0.1987)	0.2744(0.0394)	1.1176(0.3203)	0.3297(0.0570)	1.2098(0.3691)	0.3321(0.0690)
	*PWM*	0.2671(0.0655)	0.0728(0.0202)	0.6260(0.1855)	0.1662(0.0384)	0.9558(0.2908)	0.2533(0.0535)	1.0767(0.2773)	0.2837(0.0595)
	*SDPWM*	0.6439(0.1477)	0.2448(0.0286)	1.1436(0.3521)	0.3438(0.0515)	1.4453(0.4881)	0.3859(0.0694)	1.4884(0.5198)	0.3591(0.0733)
	*POME*	0.4919(0.06761)	0.2212(0.0212)	0.8307(0.1890)	0.2938 (0.0385)	1.0809(0.2936)	0.3395(0.0544)	1.1712(0.3413)	0.3429(0.0663)
n = 50	*MLE*	0.1796(0.0063)	0.0795(0.0027)	0.3091(0.0178)	0.1078(0.0051)	0.4499(0.0366)	0.1348(0.0078)	0.5772(0.0580)	0.1559(0.0109)
	*MOM*	0.1689(0.0067)	0.0671(0.0029)	0.3157(0.0203)	0.0849(0.0053)	0.4918(0.0442)	0.1402(0.0088)	0.6362(0.0679)	0.1655(0.0122)
	*PWM*	0.1031(0.0059)	0.0228(0.0025)	0.2777(0.0169)	0.0110(0.0048)	0.4164(0.0365)	0.1112(0.0076)	0.4843(0.0532)	0.1327(0.0108)
	*SDPWM*	0.2137(0.0096)	0.0851(0.0033)	0.4851(0.0394)	0.1073(0.0075)	0.6841(0.0755)	0.1838(0.0118)	0.8264(0.0990)	0.1988(0.0155)
	*POME*	0.1803(0.0063)	0.0807(0.0027)	0.3094(0.0178)	0.0951(0.0051)	0.4536(0.0372)	0.1376(0.0079)	0.5836(0.0591)	0.1599(0.0111)
n = 100	*MLE*	0.0759(0.0011)	0.0330(0.0006)	0.1303 (0.0031)	0.0446(0.0011)	0.1885(0.0062)	0.0561(0.0017)	0.2535(0.0111)	0.0675(0.0025)
	*MOM*	0.0713(0.0012)	0.0263(0.0006)	0.1702 (0.0035)	0.0434(0.0011)	0.2114(0.0076)	0.0596(0.0019)	0.3018(0.0141)	0.0777(0.0028)
	*PWM*	0.0506(0.0010)	0.0115(0.0005)	0.1289 (0.0030)	0.0423(0.0010)	0.1793(0.0060)	0.0472(0.0017)	0.2324(0.0109)	0.0543(0.0024)
	*SDPWM*	0.0918(0.0016)	0.0356(0.0007)	0.1931(0.0058)	0.0621 (0.0015)	0.3331(0.0147)	0.0930(0.0028)	0.4342(0.0246)	0.1050(0.0039)
	*POME*	0.0767(0.0011)	0.0339(0.0006)	0.1305(0.0032)	0.0459(0.0011)	0.1899(0.0063)	0.0574(0.0017)	0.2574(0.0112)	0.0698(0.0025)
n = 300	*MLE*	0.0275(0.0001)	0.0175(0.0001)	0.0483(0.0003)	0.0243(0.0001)	0.0718(0.0006)	0.0310(0.0002)	0.0976(0.0010)	0.0377(0.0003)
	*MOM*	0.0255(0.0001)	0.0158(0.0001)	0.0476(0.0003)	0.0234(0.0001)	0.0694(0.0007)	0.0284(0.0002)	0.0960(0.0013)	0.0344(0.0003)
	*PWM*	0.0243(0.0001)	0.0151(0.0001)	0.0449(0.0003)	0.0218(0.0001)	0.0600(0.0006)	0.0247(0.0002)	0.0795(0.0010)	0.0290(0.0003)
	*SDPWM*	0.0269(0.0001)	0.0160(0.0001)	0.0524(0.0004)	0.0237(0.0001)	0.0818(0.0010)	0.0304(0.0002)	0.0991(0.0017)	0.0307(0.0004)
	*POME*	0.0271(0.0001)	0.0172(0.0001)	0.0471(0.0003)	0.0236(0.0001)	0.0700(0.0006)	0.0300(0.0002)	0.0955(0.0010)	0.0364(0.0003)

**Table 6:** Bias and MSE (parentheses) for the estimators of *α* and *β* when *α* < *β* and for different choices of *n*.

		*α* = 2, *β* = 3		*α* = 2, *β* = 5		*α* = 3, *β* = 5		*α* = 4, *β* = 5	
		$\hat{\alpha }$	$\hat{\beta }$	$\hat{\alpha }$	$\hat{\beta }$	$\hat{\alpha }$	$\hat{\beta }$	$\hat{\alpha }$	$\hat{\beta }$
n = 20	*MLE*	0.4962(0.0752)	0.3336(0.0480)	0.4960(0.0751)	0.5551(0.1327)	0.8394(0.1914)	0.4932(0.1068)	1.0929(0.2936)	0.4269(0.0849)
	*MOM*	0.5067(0.0833)	0.3178(0.0506)	0.5845(0.0989)	0.6028(0.1535)	0.9348(0.2306)	0.5149(0.1198)	1.1690(0.3358)	0.4315(0.0922)
	*PWM*	0.3228(0.0713)	0.1625(0.0476)	0.4316(0.0735)	0.2365(0.1314)	0.7509(0.1885)	0.3957(0.1060)	0.9949(0.2929)	0.03517(0.0818)
	*SDPWM*	0.7404(0.1835)	0.4032(0.0688)	0.8512(0.2209)	0.7024(0.2058)	1.3077(0.4181)	0.6344(0.1553)	1.4947(0.5046)	0.4936(0.1092)
	*POME*	0.4916(0.0740)	0.3316(0.0486)	0.4908(0.0746)	0.5755(0.1402)	0.8297(0.1888)	0.4882(0.1068)	1.0835(0.3413)	0.4239(0.0849)
n = 50	*MLE*	0.1796(0.0063)	0.1192(0.0056)	0.1796(0.0063)	0.1986(0.0161)	0.3091(0.0188)	0.1797(0.0134)	0.4499(0.0372)	0.1685(0.0122)
	*MOM*	0.1916(0.0075)	0.1182(0.0064)	0.2282(0.0090)	0.2385(0.0198)	0.3723(0.0242)	0.2069(0.0163)	0.5265(0.0476)	0.1891(0.0144)
	*PWM*	0.1425(0.0062)	0.0745(0.0055)	0.1759(0.0060)	0.1795(0.0156)	0.2972(0.0180)	0.1597(0.0133)	0.4277(0.0366)	0.1479(0.0119)
	*SDPWM*	0.2497(0.0121)	0.1497(0.0082)	0.2970(0.0163)	0.2901(0.0256)	0.5355(0.0476)	0.2773(0.0229)	0.7187(0.0799)	0.2400(0.0189)
	*POME*	0.1803(0.0063)	0.1210(0.0057)	0.1800(0.0063)	0.2013(0.0165)	0.3103(0.0188)	0.1827(0.0135)	0.4530(0.0376)	0.1718(0.0122)
n = 100	*MLE*	0.0759(0.0012)	0.0496(0.0013)	0.0759(0.0012)	0.0826(0.0033)	0.1289(0.0031)	0.0743(0.0029)	0.1885(0.0062)	0.0701(0.0027)
	*MOM*	0.0838(0.0014)	0.0495(0.0013)	0.1031(0.0016)	0.1058(0.0041)	0.1629(0.0041)	0.0901(0.0034)	0.2294(0.0082)	0.0822(0.0031)
	*PWM*	0.0749(0.0011)	0.0452(0.0012)	0.07110(0.0012)	0.0769(0.0030)	0.1253(0.0030)	0.0654(0.0028)	0.1784(0.0060)	0.0593(0.0026)
	*SDPWM*	0.1067(0.0019)	0.0641(0.0016)	0.1257(0.0022)	0.1278(0.0049)	0.2411(0.0074)	0.1326(0.0047)	0.3497(0.0154)	0.1223(0.0044)
	*POME*	0.0766(0.0012)	0.0507(0.0013)	0.0765(0.0011)	0.0842(0.0033)	0.1298(0.0031)	0.0761(0.0030)	0.1906(0.0063)	0.0723(0.0027)
n = 300	*MLE*	0.0275(0.0001)	0.0262(0.0001)	0.0289(0.0001)	0.0436(0.0004)	0.0483(0.0003)	0.0405(0.0003)	0.0718(0.0006)	0.0388(0.0003)
	*MOM*	0.0258(0.0001)	0.0239(0.0001)	0.0275(0.0001)	0.0421(0.0004)	0.0492(0.0004)	0.0383(0.0004)	0.0716(0.0008)	0.0359(0.0003)
	*PWM*	0.0256(0.0001)	0.0230(0.0001)	0.0270(0.0001)	0.0417(0.0003)	0.0421(0.0003)	0.0340(0.0003)	0.0614(0.0006)	0.0316(0.0003)
	*SDPWM*	0.0287(0.0002)	0.0252(0.0002)	0.0323(0.0002)	0.0460(0.0005)	0.0606(0.0005)	0.0450(0.0003)	0.0779(0.0010)	0.0348(0.0004)
	*POME*	0.0271(0.0001)	0.0257(0.0001)	0.0293(0.0001)	0.0426(0.0004)	0.0471(0.0003)	0.0393(0.0003)	0.0691(0.0006)	0.0370(0.0003)

**Table 7:** *Bias* and *MSE* (parentheses) for the estimators of *α* and *β* when *α* > *β* and for different choices of *n*.

		*α* = 3, *β* = 2		*α* = 4, *β* = 3		*α* = 5, *β* = 2		*α* = 5, *β* = 4	
		$\hat{\alpha }$	$\hat{\beta }$	$\hat{\alpha }$	$\hat{\beta }$	$\hat{\alpha }$	$\hat{\beta }$	$\hat{\alpha }$	$\hat{\beta }$
n = 20	*MLE*	0.8394(0.1914)	0.1381(0.0106)	1.0929(0.2936)	0.2561(0.0306)	1.1873(0.3420)	0.1381(0.0106)	1.1889(0.3420)	0.2762(0.0426)
	*MOM*	0.733(0.1826)	0.1171(0.0102)	1.0611(0.3021)	0.2346(0.0308)	1.1035(0.3345)	0.1171(0.0102)	1.1674(0.3564)	0.2546(0.0459)
	*PWM*	0.4192(0.1386)	0.0047(0.0087)	0.6230(0.2420)	0.0942(0.0294)	0.6688(0.2998)	0.0380(0.0100)	0.7974(0.3229)	0.1347(0.0422)
	*SDPWM*	1.0168(0.2970)	0.1180(0.0112)	1.3771(0.4621)	0.2800(0.0384)	1.2637(0.4303)	0.1279(0.0119)	1.4499(0.5043)	0.2847(0.0506)
	*POME*	0.8295(0.1880)	0.1401(0.0108)	1.0786(0.2895)	0.2548(0.0306)	1.1756(0.3411)	0.1401(0.0108)	1.1721(0.3399)	0.2766(0.0429)
n = 50	*MLE*	0.3091(0.0180)	0.0719(0.0021)	0.4499(0.0366)	0.1011(0.0043)	0.5772(0.0580)	0.0624(0.0017)	0.5772(0.0580)	0.1247(0.0071)
	*MOM*	0.5314(0.0185)	0.0598(0.0022)	0.4515(0.0402)	0.0950(0.0046)	0.5314(0.0580)	0.0519(0.0017)	0.6003(0.0641)	0.1235(0.0075)
	*PWM*	0.2240(0.0164)	0.0015(0.0021)	0.2519(0.0345)	0.0266(0.0044)	0.2909(0.0523)	0.0071(0.0017)	0.3065(0.0542)	0.0292(0.0069)
	*SDPWM*	0.6393(0.0298)	0.0793(0.0029)	0.6277(0.0690)	0.1265(0.0064)	0.5992(0.0715)	0.0567(0.0021)	0.7925(0.0984)	0.1538(0.0098)
	*POME*	0.5774(0.0180)	0.0732(0.0022)	0.4514(0.0367)	0.1029(0.0044)	0.5769(0.0579)	0.0634(0.0018)	0.5835(0.0590)	0.1280(0.0071)
n = 100	*MLE*	0.1289(0.0030)	0.0297(0.0005)	0.1885(0.0062)	0.0420(0.0009)	0.2535(0.0109)	0.0270(0.0004)	0.2535(0.0109)	0.0540(0.0016)
	*MOM*	0.1166(0.0032)	0.0229(0.0005)	0.1903(0.0069)	0.0390(0.0010)	0.2286(0.0110)	0.0212(0.0004)	0.2790(0.0129)	0.0565(0.0017)
	*PWM*	0.0563(0.0030)	0.0003(0.0005)	0.1073(0.0062)	0.0095(0.0009)	0.1293(0.0096)	0.0029(0.0004)	0.1687(0.0107)	0.0215(0.0015)
	*SDPWM*	0.1594(0.0047)	0.0330 (0.0006)	0.2914(0.0127)	0.0601(0.0014)	0.2799(0.0150)	0.0257(0.0005)	0.4295(0.0237)	0.0853(0.0025)
	*POME*	0.1304(0.0031)	0.0306(0.0005)	0.1913(0.0063)	0.0435(0.0009)	0.2566(0.0111)	0.0278(0.0004)	0.2542(0.0111)	0.0549(0.0016)
n = 300	*MLE*	0.0483(0.0003)	0.0162(0.0000)	0.0718(0.0006)	0.0233(0.0001)	0.0976(0.0011)	0.0151(0.0000)	0.0976(0.0011)	0.0302(0.0002)
	*MOM*	0.0440(0.0003)	0.0145(0.0001)	0.0672(0.0007)	0.0211(0.0001)	0.093(0.0011)	0.0142(0.0000)	0.0942(0.0012)	0.0276(0.0002)
	*PWM*	0.0285(0.0003)	0.0083(0.0000)	0.0551(0.0006)	0.0165(0.0001)	0.0769(0.0010)	0.0112(0.0000)	0.0847(0.0011)	0.0246(0.0002)
	*SDPWM*	0.0473(0.0004)	0.0145(0.0001)	0.0766(0.0009)	0.0217(0.0001)	0.0886(0.0012)	0.0128(0.0000)	0.1103(0.0017)	0.0293(0.0002)
	*POME*	0,0472(0.0003)	0.0158(0.0000)	0.0697(0.0006)	0.0224(0.0001)	0.0947(0.0011)	0.0145(0.0000)	0.0941(0.0011)	0.0287(0.0002)

**Table 8:** The average length (L) and coverage probability (CP) of the bootstrap confidence intervals for *α* and *β* when *α* = *β* and for different choices of *n*.

		*α* = *β* = 2				*α* = *β* = 3				*α* = *β* = 4				*α* = *β* = 5			
		*α*		*β*		*α*		*β*		*α*		*β*		*α*		*β*	
		*L*	*CP*	*L*	*CP*	*L*	*CP*	*L*	*CP*	*L*	*CP*	*L*	*CP*	*L*	*CP*	*L*	*CP*
n = 100	*MLE*	1.323	0.912	0.909	0.927	2.213	0.913	1.254	0.927	3.179	0.914	1.595	0.929	4.058	0.917	1.925	0.930
	*MOM*	1.399	0.914	0.934	0.926	2.371	0.919	1.302	0.922	3.475	0.920	1.678	0.931	4.418	0.929	2.017	0.933
	*PWM*	1.323	0.915	0.902	0.936	2.211	0.920	1.252	0.936	3.160	0.925	1.510	0.938	4.044	0.933	1.901	0.962
	*SDPWM*	1.593	0.913	1.009	0.927	2.987	0.918	1.490	0.931	4.478	0.919	1.959	0.933	5.380	0.930	2.323	0.935
	*POME*	1.325	0.913	0.909	0.931	2.214	0.911	1.264	0.932	3.190	0.916	1.610	0.933	4.058	0.915	1.924	0.934
n = 300	*MLE*	0.702	0.930	0.507	0.931	1.148	0.929	0.702	0.938	1.656	0.926	0.903	0.938	2.191	0.924	1.097	0.937
	*MOM*	0.740	0.920	0.521	0.930	1.231	0.933	0.728	0.929	1.794	0.925	0.946	0.937	2.424	0.931	1.169	0.936
	*PWM*	0.697	0.937	0.505	0.939	1.147	0.936	0.702	0.941	1.640	0.935	0.895	0.944	2.165	0.943	1.085	0.958
	*SDPWM*	0.812	0.933	0.557	0.934	1.441	0.926	0.815	0.936	2.217	0.921	1.097	0.944	3.097	0.939	1.388	0.941
	*POME*	0.703	0.920	0.507	0.936	1.158	0.922	0.707	0.939	1.656	0.919	0.903	0.938	2.191	0.921	1.097	0.941
n = 500	*MLE*	0.534	0.937	0.389	0.937	0.877	0.936	0.543	0.941	1.249	0.936	0.690	0.941	1.646	0.939	0.843	0.940
	*MOM*	0.566	0.938	0.407	0.943	0.871	0.940	0.567	0.940	1.369	0.933	0.734	0.942	1.844	0.937	0.906	0.942
	*PWM*	0.530	0.941	0.389	0.944	0.840	0.945	0.541	0.948	1.241	0.940	0.684	0.947	1.611	0.949	0.836	0.955
	*SDPWM*	0.614	0.937	0.428	0.938	1.080	0.938	0.625	0.948	1.644	0.937	0.839	0.955	2.299	0.945	1.057	0.959
	*POME*	0.531	0.937	0.390	0.944	0.871	0.936	0.543	0.944	1.244	0.937	0.694	0.944	1.642	0.935	0.843	0.944
n = 1000	*MLE*	0.372	0.940	0.274	0.952	0.610	0.938	0.382	0.954	0.869	0.939	0.487	0.945	1.154	0.943	0.597	0.944
	*MOM*	0.395	0.941	0.286	0.947	0.653	0.944	0.399	0.943	0.949	0.939	0.516	0.944	1.275	0.941	0.637	0.945
	*PWM*	0.363	0.945	0.266	0.949	0.599	0.953	0.374	0.950	0.865	0.943	0.481	0.949	1.144	0.953	0.591	0.947
	*SDPWM*	0.429	0.942	0.302	0.946	0.750	0.960	0.440	0.960	1.136	0.939	0.590	0.970	1.575	0.960	0.746	0.972
	*POME*	0.373	0.941	0.276	0.949	0.613	0.938	0.384	0.948	0.875	0.939	0.491	0.947	1.154	0.943	0.597	0.948

**Table 9:** The average length (L) and coverage probability (CP) of the bootstrap confidence intervals for *α* and *β* when *α* < *β* and for different choices of *n*.

		*α* = 2, *β* = 3				*α* = 2, *β* = 5				*α* = 3, *β* = 5				*α* = 4, *β* = 5			
		$\hat{\alpha }$		$\hat{\beta }$		$\hat{\alpha }$		$\hat{\beta }$		$\hat{\alpha }$		$\hat{\beta }$		$\hat{\alpha }$		$\hat{\beta }$	
		*L*	*CP*	*L*	*CP*	*L*	*CP*	*L*	*CP*	*L*	*CP*	*L*	*CP*	*L*	*CP*	*L*	*CP*
n = 100	*MLE*	1.325	0.912	1.354	0.927	1.324	0.912	2.271	0.927	2.213	0.914	2.099	0.927	3.179	0.913	1.993	0.929
	*MOM*	1.473	0.913	1.428	0.931	1.606	0.909	2.494	0.924	2.582	0.926	2.273	0.929	3.603	0.928	2.141	0.931
	*PWM*	1.323	0.918	1.353	0.936	1.322	0.926	2.256	0.936	2.210	0.930	2.089	0.939	3.169	0.931	1.990	0.933
	*SDPWM*	1.700	0.913	1.551	0.929	1.836	0.913	2.681	0.931	3.289	0.912	2.606	0.932	4.670	0.944	2.505	0.945
	*POME*	1.324	0.913	1.363	0.931	1.325	0.913	2.271	0.931	2.214	0.911	2.107	0.936	3.190	0.915	2.013	0.933
n = 300	*MLE*	0.703	0.930	0.760	0.931	0.697	0.930	1.261	0.933	1.148	0.929	1.170	0.933	1.640	0.926	1.119	0.936
	*MOM*	0.773	0.931	0.798	0.932	0.833	0.912	1.391	0.929	1.323	0.938	1.274	0.936	1.855	0.931	1.210	0.939
	*PWM*	0.697	0.943	0.757	0.939	0.609	0.939	1.260	0.939	1.146	0.939	1.168	0.944	1.638	0.938	1.117	0.938
	*SDPWM*	0.847	0.920	0.849	0.929	0.894	0.921	1.456	0.935	1.547	0.923	1.416	0.937	2.292	0.956	1.400	0.959
	*POME*	0.702	0.920	0.760	0.936	0.703	0.921	1.266	0.936	1.158	0.923	1.178	0.939	1.656	0.920	1.129	0.938
n = 500	*MLE*	0.534	0.937	0.583	0.937	0.534	0.937	0.972	0.937	0.877	0.936	0.902	0.938	1.249	0.936	0.869	0.937
	*MOM*	0.590	0.931	0.619	0.942	0.635	0.921	1.076	0.937	1.009	0.940	0.986	0.941	1.415	0.941	0.978	0.941
	*PWM*	0.529	0.946	0.584	0.944	0.526	0.942	0.970	0.944	0.862	0.942	0.901	0.946	1.243	0.944	0.865	0.944
	*SDPWM*	0.639	0.936	0.652	0.945	0.671	0.937	1.117	0.943	1.153	0.937	1.085	0.945	1.704	0.966	1.173	0.963
	*POME*	0.530	0.937	0.584	0.944	0.529	0.937	0.973	0.944	0.871	0.937	0.905	0.944	1.249	0.938	0.877	0.940
n = 1000	*MLE*	0.372	0.939	0.411	0.952	0.372	0.943	0.685	0.952	0.610	0.938	0.636	0.954	0.609	0.953	0.862	0.939
	*MOM*	0.412	0.937	0.435	0.944	0.442	0.928	0.756	0.940	0.699	0.942	0.694	0.946	0.659	0.945	0.939	0.944
	*PWM*	0.373	0.952	0.412	0.949	0.373	0.948	0.689	0.949	0.609	0.948	0.621	0.949	0.604	0.948	0.860	0.948
	*SDPWM*	0.446	0.941	0.459	0.953	0.467	0.944	0.786	0.958	0.797	0.938	0.762	0.953	0.754	0.969	1.074	0.969
	*POME*	0.373	0.941	0.413	0.949	0.373	0.942	0.689	0.949	0.613	0.939	0.641	0.946	0.614	0.948	0.865	0.944

**Table 10:** The average length (L) and coverage probability (CP) of the bootstrap confidence intervals for *α* and *β* when *α* < *β* and for different choices of *n*.

		*α* = 3, *β* = 2				*α* = 4, *β* = 2				*α* = 5, *β* = 2				*α* = 5, *β* = 4			
		$\hat{\alpha }$		$\hat{\beta }$		$\hat{\alpha }$		$\hat{\beta }$		$\hat{\alpha }$		$\hat{\beta }$		$\hat{\alpha }$		$\hat{\beta }$	
		*L*	*CP*	*L*	*CP*	*L*	*CP*	*L*	*CP*	*L*	*CP*	*L*	*CP*	*L*	*CP*	*L*	*CP*
n = 100	*MLE*	2.211	0.913	0.836	0.927	3.179	0.914	0.797	0.929	4.044	0.917	0.761	0.930	4.056	0.919	1.140	0.930
	*MOM*	2.270	0.914	0.855	0.923	3.225	0.915	0.810	0.928	4.086	0.917	0.771	0.929	4.175	0.920	1.165	0.928
	*PWM*	2.040	0.919	0.823	0.933	3.171	0.924	0.709	0.935	4.040	0.927	0.760	0.938	4.043	0.937	1.139	0.939
	*SDPWM*	2.723	0.911	0.954	0.929	3.901	0.915	0.916	0.933	4.727	0.916	0.860	0.935	5.021	0.918	1.334	0.934
	*POME*	2.214	0.911	0.843	0.932	3.191	0.914	0.805	0.933	4.056	0.916	0.770	0.936	4.056	0.918	1.155	0.933
n = 300	*MLE*	1.148	0.929	0.468	0.938	1.640	0.926	0.448	0.938	2.165	0.924	0.434	0.937	2.162	0.925	0.651	0.937
	*MOM*	1.187	0.932	0.478	0.933	1.677	0.920	0.456	0.933	2.198	0.929	0.442	0.932	2.258	0.932	0.667	0.931
	*PWM*	1.133	0.938	0.461	0.938	1.557	0.936	0.442	0.941	2.159	0.941	0.433	0.941	2.157	0.939	0.648	0.943
	*SDPWM*	1.355	0.922	0.527	0.936	1.958	0.924	0.511	0.936	2.610	0.940	0.501	0.939	2.817	0.940	0.785	0.948
	*POME*	1.158	0.924	0.471	0.935	1.657	0.920	0.452	0.937	2.191	0.921	0.439	0.939	2.191	0.921	0.658	0.939
n = 500	*MLE*	0.877	0.936	0.361	0.954	1.249	0.939	0.345	0.944	1.646	0.934	0.334	0.940	1.646	0.924	0.502	0.942
	*MOM*	0.906	0.941	0.374	0.945	1.279	0.940	0.355	0.945	1.679	0.942	0.344	0.945	1.722	0.932	0.529	0.942
	*PWM*	0.865	0.943	0.359	0.951	1.244	0.942	0.343	0.948	1.633	0.944	0.331	0.946	1.639	0.940	0.499	0.949
	*SDPWM*	1.023	0.940	0.405	0.948	1.473	0.944	0.392	0.948	1.954	0.957	0.384	0.959	2.097	0.939	0.601	0.962
	*POME*	0.871	0.937	0.362	0.948	1.245	0.937	0.347	0.944	1.643	0.937	0.337	0.943	1.643	0.921	0.506	0.944
n = 1000	*MLE*	0.610	0.939	0.254	0.948	0.869	0.936	0.243	0.937	1.144	0.942	0.236	0.955	1.144	0.942	0.354	0.955
	*MOM*	0.631	0.943	0.263	0.947	0.889	0.943	0.250	0.946	1.166	0.944	0.242	0.950	1.193	0.933	0.366	0.945
	*PWM*	0.599	0.947	0.250	0.951	0.865	0.939	0.246	0.944	1.139	0.949	0.233	0.950	1.143	0.940	0.351	0.951
	*SDPWM*	0.712	0.954	0.285	0.957	1.020	0.965	0.276	0.963	1.341	0.963	0.269	0.962	1.444	0.960	0.422	0.969
	*POME*	0.613	0.938	0.256	0.948	0.875	0.939	0.246	0.944	1.155	0.941	0.239	0.947	1.154	0.937	0.358	0.947

Tables 5–7 show the *Bias* and *MSE* values for each estimating method. It is, therefore, critical to understand how each estimating method handles interval estimation. As a consequence, at 95% confidence levels, we generated parametric Bootstrap confidence intervals and assessed the coverage probability and average length for these intervals. Tables 8–10 present a summary of our findings. From these tables, the following conclusions may be inferred.

- The interval average length is narrower and the coverage rate is higher as sample size *n* increases.
- The average length and coverage probability of the *MLE* are not satisfactory, whereas the *PWM* outperforms all other methods by providing the shortest average length and the highest coverage probability that is close to the nominal value.
- In terms of average length and coverage probability, all estimating methods for the shape parameter *β* are comparable. However, this is not the case, when estimating *α*.

## Conclusions and remarks

In this paper, we have considered the *MLE*, *MOM*, *PWM*, *SD*-*PWM* and the *POME* to derive estimates for the shape parameters of the Kumaraswamy distribution. We conducted an extensive simulation analysis to compare these approaches with various sample sizes and unknown parameter combinations. The *Bias*, *MSE* and Bootstrap confidence interval length and coverage probability have been obtained. The simulation findings, as well as the results from the two real data sets, demonstrated that the *PWM* unequivocally outperforms all other estimating methods. Furthermore, *POME* and *MLE* are identical in their parameter estimates.

## Supporting information

S1 File (PDF)
