# Supplementary material for: The principle of maximum entropy and the probability-weighted moments for estimating the parameters of the Kumaraswamy distribution
Source: PLoS One. 2022 May 31;17(5):e0268602. doi: 10.1371/journal.pone.0268602 (PMC9154196; doi:10.1371/journal.pone.0268602)
Supplement: S1 File — (PDF) [file pone.0268602.s001.pdf]

Real Data I

The data for this application were obtained in a civil engineering context. It represents the maximum flood level (in millions of cubic feet per second) for the Susquehanna River at Harrisburg, Pennsylvania. The numbers in this data represent the maximum flood level for four years, the first number being 0.654 for the period 1890-1893, and the last one being 0.265, which is for the time period 1966-1969. The data were utilized by [28] and it is given in the table below.

0.654	0.613	0.315	0.449	0.297	0.402	0.379	0.423	0.379	0.324
0.269	0.740	0.418	0.412	0.494	0.416	0.338	0.392	0.484	0.265

Real Life Data 2:

Our second data set were taken from a clinical trial aimed at testing the efficacy of an analgesic. In the table below, relief times (in hours) are shown for 50 arthritic patients treated with a fixed dosage of this medication. These data were first utilized by [30] and later by [31].

0.70	0.84	0.58	0.50	0.55	0.82	0.59	0.71	0.72	0.61
0.62	0.49	0.54	0.36	0.36	0.71	0.35	0.64	0.84	0.55
0.59	0.29	0.75	0.46	0.46	0.60	0.60	0.36	0.52	0.68
0.80	0.55	0.84	0.34	0.34	0.70	0.49	0.56	0.71	0.61
0.57	0.73	0.75	0.44	0.44	0.81	0.80	0.87	0.29	0.50

- [28] Dumonceaux, Robert, and Charles E. Antle. Discrimination between the log-normal and the Weibull distributions. Technometrics 15, no. 4 (1973): 923-926.
- [30] Wingo, D. R. (1983). Maximum likelihood methods for fitting the Burr type XII distribution to life test data. Biometrical journal, 25(1), 77-84.
- [31] Soliman, A. A., Abd Ellah, A. H., Abou-Elheggag, N. A., & Modhesh, A. A. (2012). Estimation of the coefficient of variation for non-normal model using progressive first-failure-censoring data. Journal of Applied Statistics, 39(12), 2741-2758.
